# Revisiting Enzyme Replacement Therapy for Aspartylglucosaminuria: Truncated Phosphotransferase Enhances Mannose‐6‐Phosphorylation and Cellular Uptake of Aspartylglucosaminidase

**DOI:** 10.1002/jimd.70248

**Published:** 2026-09-01

**Authors:** Antje Banning, Lidia Reznikova, Adla Murad, Ritva Tikkanen

**Affiliations:** ^1^ Institute of Biochemistry, Medical Faculty University of Giessen Giessen Germany

**Keywords:** aspartylglucosaminuria, enzyme replacement therapy, lysosomal storage disorders, mannose‐6‐phosphate, N‐glycosylation, recombinant proteins

## Abstract

Aspartylglucosaminuria (AGU) is a lysosomal storage disorder caused by a deficiency of aspartylglucosaminidase (AGA), a hydrolase involved in the degradation of N‐glycosylated proteins. Currently, no approved therapies are available for AGU. Development of enzyme replacement therapy (ERT) for AGU has been hampered by the complex proteolytic processing and activation of the AGA enzyme that involves dimerization and cleavage into two subunits. Targeting of AGA into lysosomes is mainly accomplished by a mannose‐6‐phosphate receptor‐mediated pathway, and both AGA subunits contain phosphorylated N‐glycans. In this study, we have developed an overexpression system for an optimized human AGA enzyme and a truncated GlcNAc‐1‐phosphotransferase, S1S3, that is required for the mannose‐6‐phosphorylation. We here show that the Man‐6‐phosphorylation and cellular uptake of AGA are enhanced by the coexpression of S1S3, and high amounts of affinity‐tagged recombinant human AGA can be expressed in HEK293T cells and purified from the culture medium. Impairment of the N‐glycosylation of AGA results in poor uptake into cells, indicating that the Man‐6‐dependent route is the main endocytic pathway of AGA. Furthermore, for optimal uptake, both subunits of AGA need to be glycosylated and Man‐6‐phosphorylated. The results of this study enhance the understanding of the lysosomal targeting mechanisms of AGA and pave the way for the development of ERT for AGU.

## 1 Introduction

Aspartylglucosaminuria (AGU; OMIM 208400) is a rare hereditary disease that belongs to the group of lysosomal storage disorders. AGU is caused by a deficiency of the enzyme aspartylglucosaminidase (AGA; N4‐(β‐N‐acetylglucosaminyl)‐L‐asparaginase; EC 3.5.1.26), and accumulation of N‐acetylglucosamine species such as GlcNAc‐Asn in patient tissues and body fluids is observed [1, 2]. AGU is characterized by a relatively slow but progressive developmental delay. To date, over 50 pathogenic gene variants of the AGA gene have been described, and the disease‐causing variants represent a wide spectrum of mutations, including deletions and insertions, as well as missense and nonsense variants [3]. Patient‐ and family‐specific variants that may be compound heterozygous, or homozygous in case of consanguinity, are normally found in patients originating from genetic backgrounds other than Finnish. Due to a founder effect, the AGU_Fin‐major_ allele with two missense variants (c.482G>A + c.488G>C; p.Arg161Gln + Cys163Ser) is found in most AGU patients in Finland, thus representing the most common genetic variant in AGU [4, 5, 6]. These two missense substitutions are always found in *cis* in the AGU_Fin‐major_ allele, but only the Cys163Ser substitution is pathogenic, whereas the Arg161Gln is a neutral variant, as shown before [7].

AGU is a progressive neurometabolic disease, for which there is still no causal therapy after almost 60 years of its discovery [8]. In the past, experimental stem cell transplants were carried out, but in most cases, they did not lead to a long‐lasting improvement in the AGU phenotype [9, 10, 11, 12]. In cell culture and in animal experiments with AGA knockout mice, enzyme replacement therapy (ERT) and gene therapy approaches have shown convincing results [13, 14, 15, 16, 17, 18]. However, neither ERT nor gene therapy has yet been tested in clinical trials with AGU patients. We have developed a pharmacological chaperone therapy with betaine for some AGU missense variants [19], and carried out a clinical trial (EudraCT Number 2017‐000645‐48) in Finnish AGU patients, all harboring the AGU_Fin‐major_ gene variant [20]. The publication of the results of our study is still pending, but as betaine treatment can be considered as a supportive therapy, there is still a dire need for a therapy approach that would be suitable for all AGU patients.

**FIGURE 1:**
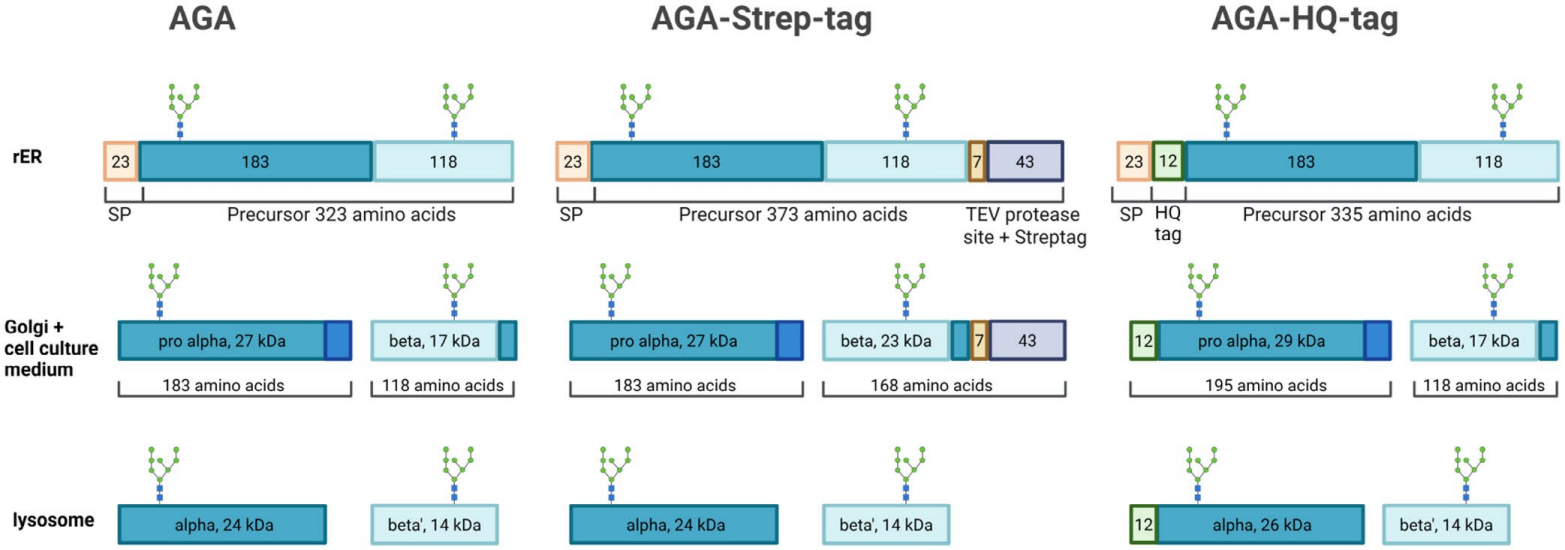
Recombinant AGA protein variants and their cellular processing. Non‐tagged, Strep‐tagged, and HQ‐tagged human AGA constructs were expressed in human cells. The processing steps within different cellular compartments and the location and presence of the tags in different compartments are shown. Created with BioRender.com. Tikkanen, R. (2026) https://BioRender.com/zd7tuc9.

The AGA enzyme is an N‐glycosylated protein synthesized in the rough endoplasmic reticulum (rER) as a precursor molecule that dimerizes and is autocatalytically cleaved into its active, heterotetrameric α2β2 form, and further proteolytic processing steps of the subunits take place in lysosomes (Figure 1). As with most soluble lysosomal hydrolases, AGA is targeted into lysosomes by means of mannose‐6‐phosphate receptor (MPR) mediated transport [21, 22]. For this, the N‐glycans of the lysosomal enzymes are enzymatically modified in the Golgi so that mannose‐6‐phosphate (M6P) residues that bind to MPR are generated. This involves the Golgi‐resident, α2β2γ2 hetero‐hexameric GlcNAc‐1‐phosphotransferase enzyme. The complexes between MPRs and lysosomal enzymes are sorted to late endosomes, from which the enzymes are then targeted to lysosomes, whereas the MPR undergoes recycling back to Golgi [23].

Mannose‐6‐phosphorylation is critical for the correct lysosomal targeting of most lysosomal enzymes. M6P is recognized in the Golgi compartment by either cation‐dependent (MPR46) or cation‐independent (MPR300) MPRs, which in turn have sorting motifs in their cytosolic tails that bind to adapter proteins in the trans Golgi network. These interactions facilitate the packing of the MPRs and their cargo into clathrin‐coated vesicles and ensure their transport to the endosomal/lysosomal system (for review see [24, 25]). This transport mechanism works for the cell's own proteins, but also for externally supplied ones, as the MPR300 is also located in the plasma membrane and can effectively endocytose lysosomal proteins. The presence of MPR300 in the plasma membrane is an important prerequisite for an effective enzyme replacement therapy (ERT), which is already available for some lysosomal storage diseases (for a review see [26, 27, 28]).

Over the past decade, there has been great progress in the development of ERT for lysosomal diseases, such as neuronal ceroid lipofuscinosis 2 (CLN2) [29], Fabry disease [30], Pompe disease [31] and others (for a review, see [27]). The therapeutic enzymes are typically purified from overexpressing eukaryotic cell lines. However, due to the activation of AGA by autoproteolysis, which requires the enzyme to be correctly folded, it has proven difficult to produce large amounts of active recombinant AGA for ERT in patients [32]. In addition, lysosomal enzymes produced at high levels in eukaryotic cells are often inefficiently M6‐phosphorylated by the endogenous GlcNAc‐1‐phosphotransferase [33, 34, 35]. Since the rate‐limiting α2β2γ2 hetero‐hexameric GlcNAc‐1‐phosphotransferase cannot be easily cloned and overexpressed due to its size and involvement of two genes, Liu et al. developed an elegant way to overcome this problem [34]. They constructed a truncated and overactive variant, S1S3, of the phosphotransferase and made it possible to co‐express the lysosomal protein of interest with the phosphotransferase. The structure of this minimal phosphotransferase that is 680 amino acids shorter and 20‐fold more active than the endogenous one has been solved by cryo‐electron microscopy [36].

The aim of this study was to develop an AGA‐overexpressing cell system from which active, M6‐phosphorylated AGA, potentially suitable for ERT, can be isolated in high quantities. We here show that HEK293T cells are suitable for the overexpression of codon‐optimized AGA that can be produced as a tagged protein, with either a cleavable Streptag at the C‐terminus or a cleavable HQ‐tag after the signal peptide. Neither tag interferes with AGA processing, activity, or uptake in patient fibroblasts. We further show that the level of M6P in AGA can be increased by co‐expressing a codon‐optimized version of the truncated S1S3 phosphotransferase. This leads to a significantly improved uptake of the recombinant AGA in patient cells. The results of our study show that large‐scale production of recombinant, M6‐phosphorylated, and active AGA is feasible, paving the way for reconsidering ERT for AGU in patients.

## 2 Material and Methods

### 2.1 Cell Culture

HEK293T (human embryonic kidney) cells were cultured in Dulbecco's modified Eagle's medium (DMEM) with high glucose, 10% fetal bovine serum (FBS), 1% penicillin/streptomycin (all from Thermo Fisher Scientific, Dreieich, Germany). AGA knockout HEK293T cells without endogenous AGA expression have been described before [37].

Primary skin fibroblasts from a homozygous AGU_Fin‐major_ patient were obtained from Coriell Institute of Medical Research (GM00568; Camden, NJ, USA). Primary skin fibroblasts from a compound heterozygous AGU patient (c.280G>C/c.503G>A; p.Gly94Arg/Trp168*) were isolated from skin punch biopsies according to our previously published method [38]. Parents provided a signed informed consent for the biopsy, fibroblast culture, storage, enzymatic and molecular analyses (approval of the ethics committee of University of Giessen, #144/21). Fibroblasts were cultured in DMEM (high glucose), 10% FBS, 1% penicillin/streptomycin, 1% non‐essential amino acids, and 1% sodium pyruvate. As controls, immortalized fibroblasts [39, 40] and primary fibroblasts GM00969 (Coriell Institute) [19] were used. All cells were grown at 8% CO_2_ and 37°C. Fibroblasts were treated for 24–72 h with increasing amounts of either purified, recombinant AGA, or with conditioned medium from AGA overexpressing cells. To block the M6P receptor on the plasma membrane, 5 mM mannose‐6‐phosphate (Cayman Chemical, Biomol, Hamburg, Germany) was added to the medium.

For some experiments, AGA overexpressing cells were treated with increasing amounts of valproic acid (Santa Cruz Biotechnology, Heidelberg, Germany). For the purification of AGA from cell culture medium, AGA overexpressing cells were grown for up to 1 week in 50 mL Cellstar Cellreactor tubes (Greiner GmbH, Frickenhausen, Germany) under constant rotation, with a starting density of 500 000 cells/mL in 20 mL culture medium supplemented with 8 mM valproic acid.

### 2.2 Plasmids and Transfections

The codon‐optimized AGA‐pcDNA3 and AGA‐pExpr‐IBA103 constructs [41] were used for transient and stable transfections in AGA‐knockout HEK293T cells. Since the Twin‐Strep‐tag encoded by pExpr‐IBA103 cannot be cleaved off, a TEV protease site was incorporated with the primers “TEV‐protease site fwd” (5′‐attttcaaggcctcgaggtcgacct‐3′) and “TEV‐protease site rev” (5′‐agaggttttcggatccgatgcaatcg‐3′), using the Q5 Site‐Directed Mutagenesis Kit (New England Biolabs, Frankfurt am Main, Germany). Similarly, an HQ‐tag was inserted in‐frame downstream of the 23 amino acids long AGA signal peptide with the primers “HQ‐tag fwd” (5′‐catcaacatcaacatcaatcctcaccgctgccc‐3′) and “HQ‐tag rev” (5′‐ttgatgttggtgttggtggcaccggaccagtgc‐3′). AGA glycosylation mutants were generated by site‐directed mutagenesis of Asn38 into Gln (N38Q) and of Thr310 into Ala (T310A), either alone or in combination. The primer sequences for the mutagenesis were as follows:

N38Q‐AGA‐opti‐fwd: GAACACCTGGCCCTTCAAGCAGGCTACCGAGGCAGCATGGA.

N38Q‐AGA‐opti‐rev: TCCATGCTGCCTCGGTAGCCTGCTTGAAGGGCCAGGTGTTC.

T310A‐AGA‐opti‐fwd: GTCATCTGTGCAAACGTGGCCGGTTCCTACGGTGCCGCCTG.

T310A‐AGA‐opti‐rev: CAGGCGGCACCGTAGGAACCGGCCACGTTTGCACAGATGAC.

For co‐expression of the truncated phosphotransferase, S1S3 (original sequence in [34]), we generated a codon‐optimized version that was synthesized and cloned by GenScript (Piscataway, NJ, USA) into pcDNA3.1(+)‐c‐Myc using KpnI/BamHI digestion. The sequence of the codon‐optimized S1S3 is available upon request.

For transient transfections, cells were seeded onto 12‐well plates 1 day before transfection. The expression constructs (500 ng of either of the AGA constructs alone, or in 1:1 combination with S1S3, or an empty vector) were transfected using MACSfectin (Miltenyi Biotec, Bergisch Gladbach, Germany) according to the manufacturer's protocol. The following day, the cells were transferred onto 6–10 cm dishes and harvested after a further 24–48 h.

### 2.3 Purification of AGA From Cell Culture Medium

AGA‐containing cell culture medium (20 mL) was centrifuged for 5 min at 250 × g, saturated with ammonium sulfate, and incubated at 4°C for 4 h. The precipitate was centrifuged for 15 min at 15 000 × g and dissolved in 1 mL PBS. Strep‐tagged AGA was then purified with either Strep‐Tactin Superflow columns or, for smaller amounts, with MagStrep “type3” XT beads (both from IBA Lifesciences GmbH, Göttingen, Germany) according to the manufacturer's protocol. The amount of purified AGA was determined after SDS‐polyacrylamide gel electrophoresis (SDS‐PAGE) with Coomassie blue staining using BSA standards. For some experiments, the Twin‐Strep‐tag was removed with TEV protease treatment for 1 h, according to the manufacturer's protocol.

### 2.4 Enzyme Activity Measurements

AGA activity in cell lysates and cell culture medium, or after purification from the culture medium was measured fluorometrically [19]. Reaction mixtures consisted of 20 μL sample (blank: lysis buffer or DMEM) and 20 μL of 50 μM Asp‐AMC (L‐Aspartic acid β‐(7‐amido‐4‐methylcoumarin) in McIlvain's phosphate–citrate buffer, pH 6.5). Samples were incubated for 2 h (overexpressing cells and purified AGA), or 24 h (fibroblasts) at 37°C, after which the reaction was terminated by adding 200 μL McIlvain's buffer, pH 4.5. All samples were measured in triplicates with a Tecan Infinite M200 plate reader (Tecan, Männedorf, Switzerland) using 355 nm excitation and 460 nm emission wavelengths.

### 2.5 Immunoprecipitation and Western Blot

For the analysis of total lysates, cells were grown in six‐well plates or 10 cm dishes and lysed in 50 mM Tris, pH 7.4, 150 mM NaCl, 2 mM EDTA, 1% Nonidet P‐40, supplemented with protease and phosphatase inhibitors. Equal protein amounts were analyzed with 15% SDS‐PAGE and Western blot on a nitrocellulose membrane. Primary antibodies were: rabbit anti‐AGA [21], mouse anti‐M6P (ABCD Antibodies SA, Genf, Switzerland), mouse anti‐Streptag (Novogene, Munich, Germany), mouse anti‐myc (Cell Signaling, Leiden, Netherlands), and mouse anti‐GAPDH (Abcam, Cambridge, UK; #ab‐8245). Detection was performed with an Odyssey XF Imaging System (LI‐COR Biotechnology, Bad Homburg, Germany).

AGA was immunoprecipitated from cell lysates of confluent 10 cm dishes. After removal of unspecific binding material with 3 × 50 μL of Pansorbin beads (Calbiochem, Merck, Darmstadt, Germany), the samples were rotated with either 5 μL of rabbit anti‐AGA antiserum or 10 μL of mouse‐anti‐M6P antiserum, coupled to 30 μL of magnetic Dynabeads Protein G and A (equal mixture) (Invitrogen, Thermo Fisher Scientific) for 16 h at 4°C. The beads were washed three times with IP‐buffer (10 mM Tris HCl, pH 8.0, 150 mM NaCl, 5 mM EDTA, 0.5% Triton X‐100). The precipitated proteins were boiled in SDS sample buffer containing 25 mM dithiothreitol, and subjected to SDS‐PAGE and Western blotting.

### 2.6 Immunofluorescence

Purified AGA was labeled with Alexa Fluor 546 Antibody Labeling Kit (Invitrogen) for 1 h at room temperature. To remove the unbound dye, the sample was purified using the column enclosed in the kit and diluted to 0.4 μg/μL. Fibroblasts were seeded on coverslips in 12‐well plates and treated with 1 μg/mL of the labeled AGA. After 24 h, the cells were fixed in 4% paraformaldehyde for 10 min at 37°C. For staining of acidic compartments, cells were incubated with rabbit anti‐LAMP1 antibodies (Abcam, Eching, Germany). Before antibody staining, the cells were permeabilized with 0.1% digitonin and blocked with 1% BSA in PBS. Staining with primary and secondary antibodies was carried out for 1 h at room temperature in PBS/1% BSA. The samples were analyzed with a Zeiss Axiovert 200 M microscope (Carl Zeiss, Oberkochen, Germany) in combination with the Aurox Clarity laser‐free, spinning‐disc confocal microscopy system (Aurox Ltd., Oxfordshire, UK).

Four independent experiments were performed and quantified using the Fiji Software [42]. Due to intensity differences, the images were first converted to binary ones, a threshold was set for the background, and the percentage of AGA‐positive area that was also LAMP‐1 positive was determined. In total, 71 cells were scored, with at least 13 cells per experiment.

### 2.7 Statistical Analysis

All experiments were performed at least three times, and the data are expressed as mean ± SD. Statistical comparisons between groups were made using Student's *t*‐tests, one‐way or two‐way ANOVA (Analysis of Variance), as appropriate, using the GraphPad Prism 5 software (San Diego, CA, USA). Values of *p* < 0.05 were considered significant (*), while values of *p* < 0.01 were considered very significant (**), and *p* < 0.001 extremely significant (***).

## 3 Results

### 3.1 Recombinant AGA Protein Production and Purification

**FIGURE 2:**
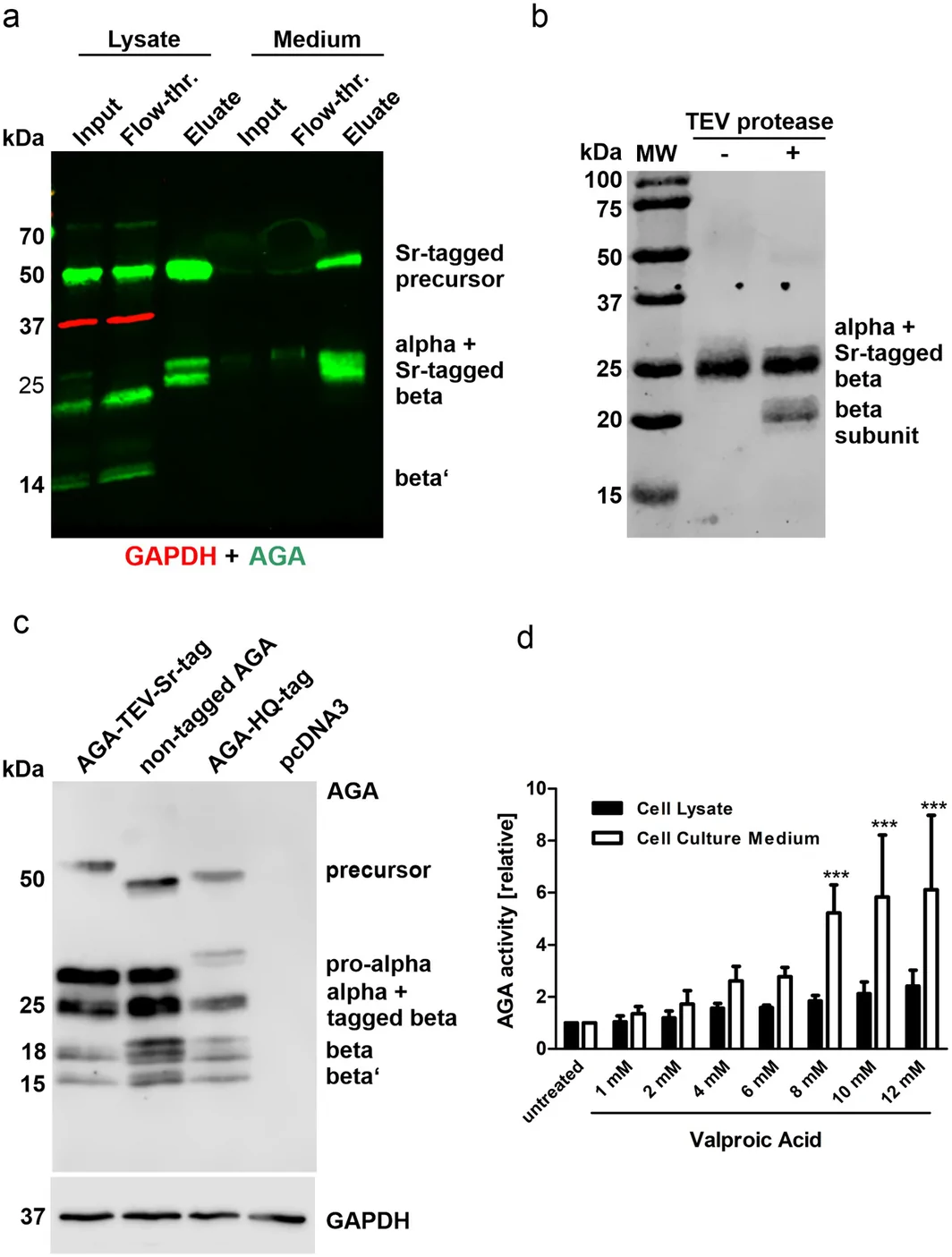
AGA protein expression, purification and tag‐removal. (a) AGA overexpressing HEK293T cells were cultured for 8 days in suspension. Strep‐tagged (Sr‐tagged) proteins were purified with Strep‐Tactin^R^ columns and analyzed by Western blot. (b) AGA purified from the medium was treated with TEV protease to remove the Streptag from the beta subunit, and tag removal was confirmed with Coomassie Blue staining after SDS‐PAGE. (c) None of the tags has a negative impact on AGA processing into alpha and beta subunits. Cell lysates from AGA overexpressing cells were analyzed by Western blot. (d) AGA overexpressing HEK293T cells were grown in six‐well plates in the presence of increasing amounts of valproic acid. After 60 h, the medium was collected, and the cells were lysed. Relative AGA activity in the medium and the lysates was measured. AGA activity in untreated cells was set as 1. *n* = 3; two‐way ANOVA. ***p < 0.001 vs. untreated.

In our previous work, we could show that codon optimization leads to a strong increase in AGA expression in HEK293T cells, as compared to overexpression using a non‐optimized sequence [41]. Therefore, we only used the codon‐optimized AGA sequence for the present study. Tagging of the AGA precursor protein with fusion tags that would facilitate its purification is challenging, due to the potentially harmful effect of the tags on the processing and activation of AGA. We designed tagged AGA constructs and tested their suitability for recombinant enzyme production. To purify AGA from cell culture medium, we introduced either a Twin‐Strep‐tag or a His‐Gln (HQ) tag (Figure 1). The Twin‐Strep‐tag was placed at the C‐terminus of AGA, and it remains as a part of the beta subunit after the precursor activation. Strep‐tagged AGA was successfully purified with Strep‐Tactin columns, and the purified AGA still carried the Streptag at the beta subunit, but it was lost after processing to the beta’ form (Figure 2a). However, since the fusion protein cloned in pExpr‐IBA‐103 vector offers no possibility of removing the tag, a cleavage site for TEV protease was inserted into the expression plasmid using targeted insertion mutagenesis (Figure 1, middle). Thus, the Twin‐Strep tag can be removed from the purified recombinant AGA (Figure 2b). In addition, a metal affinity‐based purification using an HQ‐tag provides an alternative to the Strep‐tag. Therefore, we inserted an HQ‐tag C‐terminally to the 23 amino acid‐long signal peptide of AGA, so that the tag is located at the N‐terminus of the mature alpha subunit (Figure 1, right). None of the tags had a negative effect on the processing of AGA into its alpha and beta subunits, as in all tested tagged versions of AGA, similar ratios of precursor, alpha, and beta subunits were detected (Figure 2c). The size differences are due to the different length and positions of the affinity tags.

### 3.2 Enhancement of AGA Protein Expression by Valproic Acid

Upon overexpression of AGA in mammalian cells, the endogenous sorting mechanisms to lysosomes become saturated, and large amounts of AGA protein are secreted to the cell culture medium. To increase the amount of overexpressed AGA, HEK293T cells were grown in the presence of increasing amounts of the histone deacetylase inhibitor, valproic acid (Figure 2d). The amount of active AGA in the medium was substantially enhanced by valproic acid in a dose‐dependent manner, while the intracellular AGA amount remained almost unchanged, as evidenced by measuring AGA activity (Figure 2d). These data suggest that the de novo synthesis of AGA is increased, which leads to increased secretion of the overexpressed, active protein into the culture medium. However, reduced cell growth was observed at higher concentrations of valproic acid, and 8 mM valproic acid was thus chosen for further experiments.

### 3.3 Uptake of Recombinant AGA by Patient Fibroblasts

**FIGURE 3:**
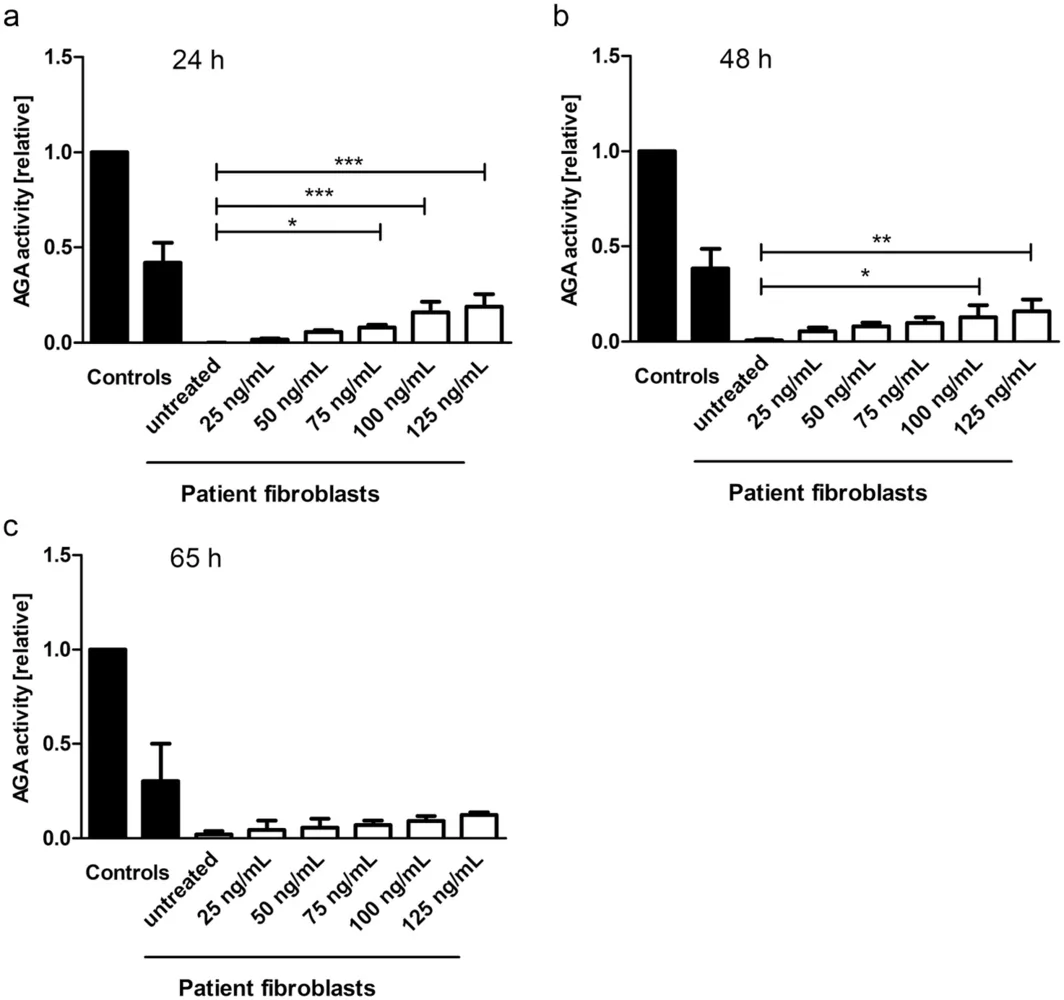
Recombinant AGA is taken up by patient fibroblasts. AGU patient fibroblasts homozygous for the AGU_Fin‐major_ variant were treated with increasing amounts of recombinant, Strep‐tagged AGA. Cells were lysed after (a) 24 h, (b) 48 h, or (c) 65 h, and the relative AGA activity in the lysates was measured. AGA activity of healthy donor fibroblasts was set as 1, to which the other values were normalized. *n* = 3; one‐way ANOVA. *p < 0.05, **p < 0.01, ***p < 0.001 vs. untreated.

AGU fibroblasts from homozygous patients with the most common pathogenic variant, AGU_Fin‐major_, were treated with increasing amounts of purified, Strep‐tagged AGA for 24 to 65 h (Figure 3). Fibroblasts from two healthy donors were used as controls. Even though the AGA activity showed a substantial degree of variation between the two healthy donor fibroblasts, only a very low AGA activity was detected in patient cells (Figure 3a–c). With increasing concentrations of recombinant AGA, the AGA enzyme activity in the patient cells increased at all incubation times, reaching 45% of the activity of the healthy donor number two after 24 h, which is very close to the expected level of an AGU carrier (50%). The half‐life of the endocytosed AGA was estimated to be at least 3 days, since after 65 h, about 60% AGA activity could be detected as compared to 24 h of uptake (Figure 3a,c).

**FIGURE 4:**
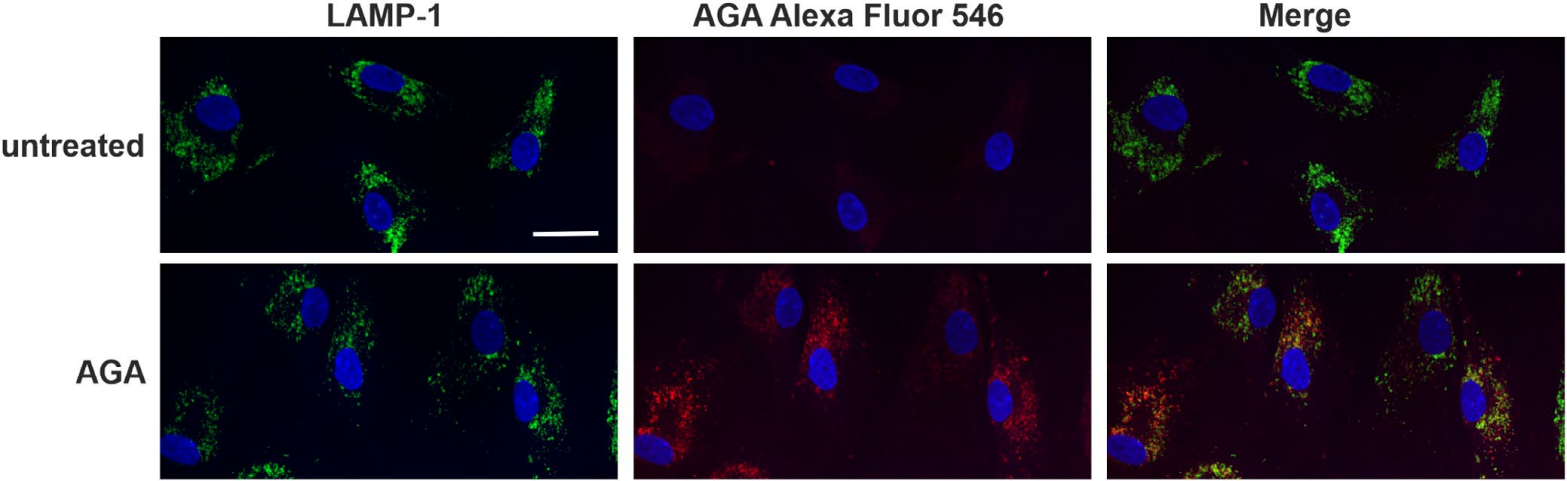
Recombinant AGA localizes in lysosomes in patient fibroblasts. AGU patient fibroblasts homozygous for the AGU_Fin‐major_ variant were grown on coverslips and either left untreated or treated with fluorescently labeled, recombinant Strep‐tagged AGA (1 μg/mL, red). After 24 h of treatment, the cells were fixed with PFA and stained for LAMP1 (green). Scale bar 20 μm. Shown is a representative experiment from four independent ones.

To show that AGA is endocytosed and targeted into lysosomes in patient cells, fluorochrome‐coupled recombinant AGA was used (Figure 4). After 24 h of uptake, recombinant AGA was clearly detectable in vesicular structures of the cells. Importantly, AGA showed a partial colocalization (54% ± 13% of AGA positive structures) with acidic, LAMP1‐positive organelles (Figure 4, lower row), indicating correct targeting of the recombinant enzyme to lysosomes. However, as the experiment was carried out as continuous uptake, the rest of the endocytosed AGA was likely still localized in earlier endocytic compartments that are not LAMP‐1 positive.

### 3.4 Mannose‐6‐Phosphorylation of the Recombinant AGA

**FIGURE 5:**
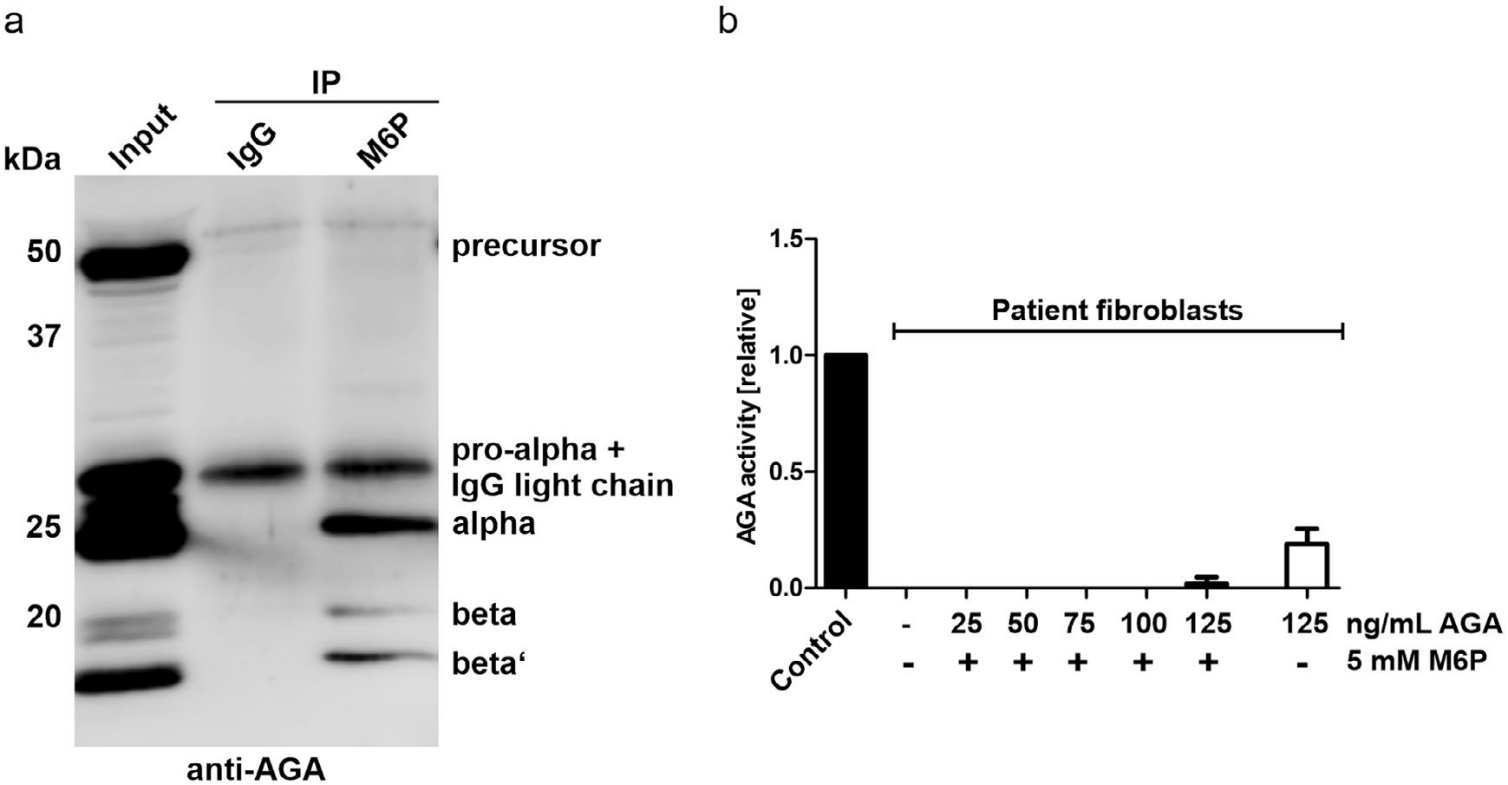
Mannose‐6‐phosphate‐dependent uptake of AGA. (a) Recombinant AGA contains mannose‐6‐phosphate. M6P containing proteins were immunoprecipitated from lysates of AGA‐overexpressing cells with an anti‐M6P antibody. As an isotype control, an unrelated IgG was used. AGA was detected by Western blot with a polyclonal rabbit anti‐AGA antibody. (b) Endocytic uptake of AGA from the medium is inhibited by 5 mM M6P. Patient fibroblasts were incubated with increasing amounts of Strep‐tagged recombinant AGA (25–125 ng/mL) in the presence of 5 mM M6P, and the relative AGA activity was determined. AGA activity of cells treated with 125 ng/mL AGA but without M6P is shown for comparison. AGA activity of healthy donor fibroblasts was set to 1 for normalization. Three independent experiments.

To investigate the importance of M6P residues for the uptake of recombinant AGA into patient cells, M6P‐carrying proteins were immunoprecipitated from cell lysates of AGA overexpressing HEK293T cells, and AGA was detected by Western blotting. The processed, active AGA carries M6P residues, whereas the AGA precursor that is found mainly in the rER is not phosphorylated (Figure 5a), consistent with the fact that M6‐phosphorylation occurs in the Golgi.

M6P‐carrying proteins are normally endocytosed by the CI‐MPR, and the recombinant AGA was taken up by this route. Simultaneous administration of AGA and 5 mM M6P to culture medium completely prevented the uptake of AGA into patient fibroblasts (Figure 5b). This indicates that M6P is essential for the uptake of recombinant AGA from the medium.

### 3.5 Increase in Mannose‐6‐Phosphorylation of AGA by S1S3 Phosphotransferase

**FIGURE 6:**
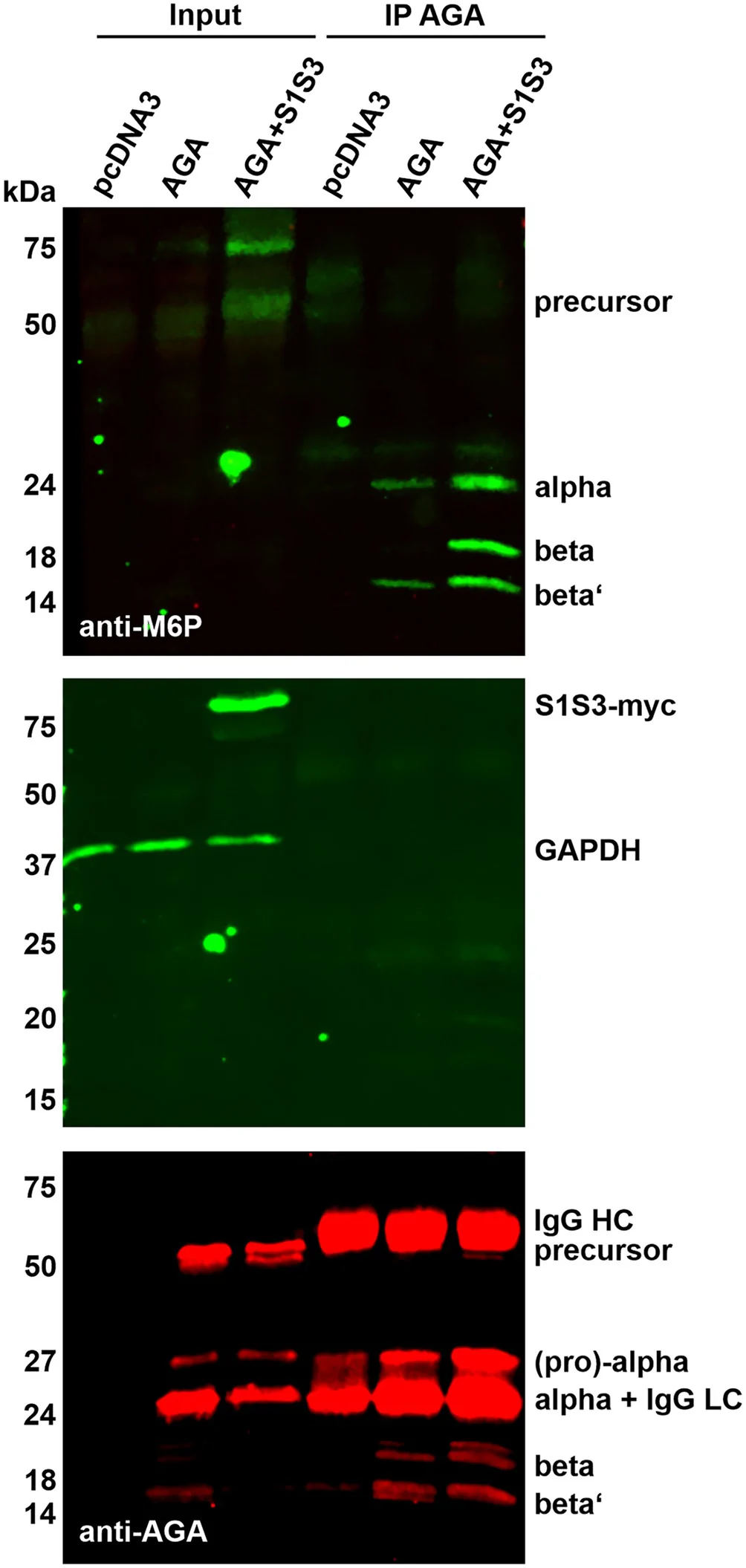
S1S3 phosphotransferase increases the amount of M6P in the recombinant AGA. AGA was immunoprecipitated from the lysates of AGA knockout cells (pcDNA3), AGA overexpressing cells (AGA), and from cells that express AGA and S1S3 (AGA + S1S3). M6P was detected by Western blot, and the overexpression of the myc‐tagged S1S3 was verified with an anti‐myc antibody in the input samples. AGA expression was confirmed with the anti‐AGA antibody that was also used for the IP, and the IgG heavy (IgG HC) and light (IgG LC) chains are thus visible in the IP samples. Equal amounts of protein in the input were verified with anti‐GAPDH. *n* = 3.

Therapeutic lysosomal enzymes that are purified from overexpressing eukaryotic cell lines are often inefficiently M6‐phosphorylated. Since AGA enters the cells in an M6P‐dependent manner, we next investigated whether the M6‐phosphorylation of recombinant AGA could be improved. For this, a codon‐optimized version of the truncated GlcNAc phosphotransferase, S1S3, was cloned as a myc‐tagged fusion protein and co‐expressed with AGA. The overexpressed AGA was then immunoprecipitated from the cell lysates, and M6‐phosphorylation was studied by Western blot. The amount of M6P on AGA could be significantly increased by co‐expression of S1S3, both at the alpha and the beta subunits (Figure 6), indicating that the endogenous phosphotransferase may not be capable of fully phosphorylating large amounts of overexpressed AGA.

**FIGURE 7:**
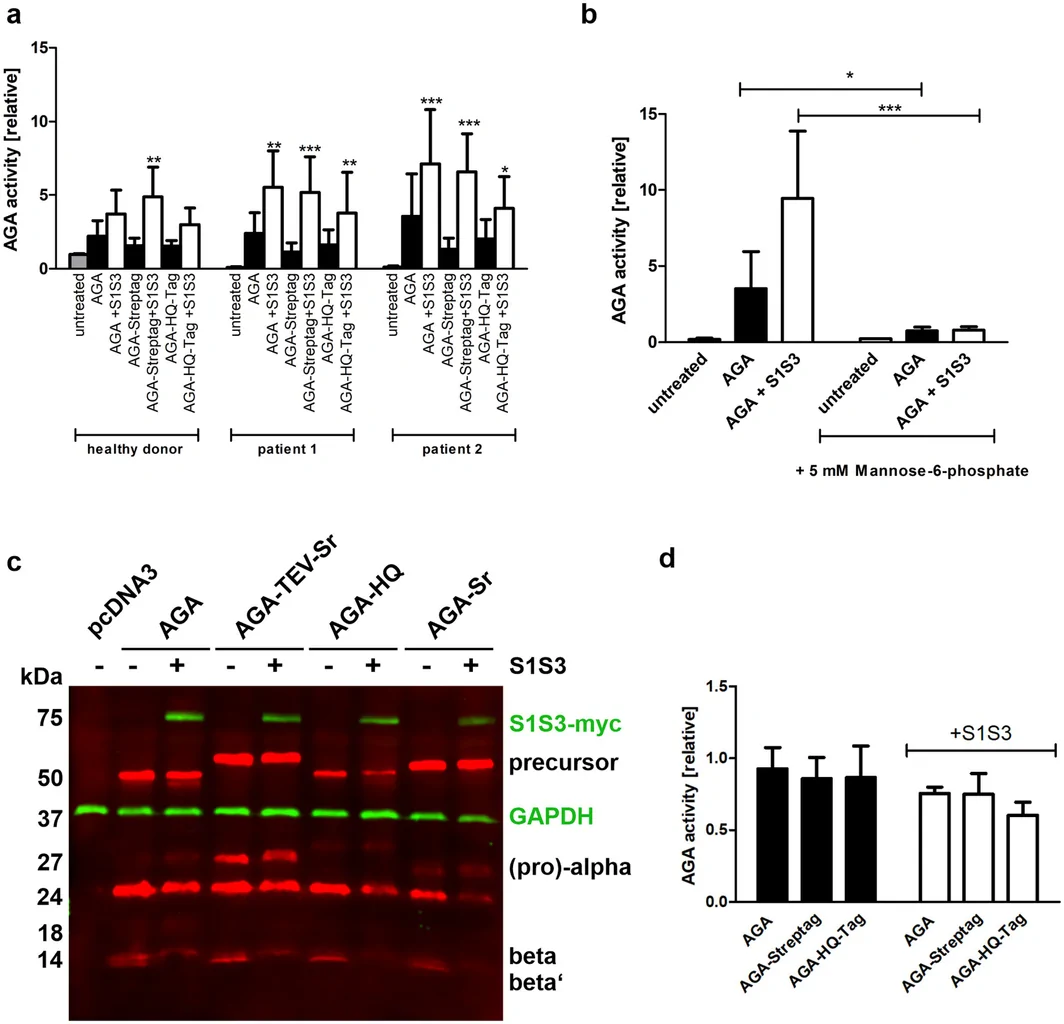
Influence of mannose‐6‐phosphorylation on AGA uptake and activity. (a) AGA (with or without TwinStreptag or HQ‐tag) was coexpressed together with S1S3 in HEK293T cells. After 48 h, the medium was collected, and the relative AGA activity was determined. Healthy donor and AGU patient fibroblasts (Patient 1: Homozygous for AGU_Fin‐major_ variant, Patient 2: Compound heterozygous for p.Gly94Arg and Trp168*) were treated for 24 h with the media containing the same amount of AGA activity. After treatment, the AGA activity was determined in the cell lysates and normalized to protein content. *n* = 5; two‐way ANOVA vs. the respective AGA without S1S3 coexpression. (b) Fibroblasts from AGU patient 2 were incubated with conditioned medium as in (a) in the presence or absence of 5 mM M6P. The relative AGA activity of healthy donor fibroblasts was set as 1, and the AGU samples were normalized to it. *n* = 4; two‐way ANOVA vs. samples without M6P. (c) Cell lysates of HEK293T cells overexpressing tagged or non‐tagged AGA and myc‐tagged S1S3 were subjected to Western blot. S1S3 expression was confirmed with anti‐myc antibodies, and equal loading with anti‐GAPDH. AGA processing into subunits was verified using an anti‐AGA antibody. Size differences of the subunits are due to the respective affinity tags. (d) Relative AGA activity was measured in cell lysates of HEK293T cells transiently transfected with Strep‐ or HQ‐tagged AGA in combination with S1S3 or an empty vector. *n* = 3; two‐way ANOVA. *p < 0.05, **p < 0.01, ***p < 0.001 for all panels.

The increased M6‐phosphorylation by overexpressed S1S3‐phosphotransferase is also functionally relevant, as coexpression with S1S3 significantly increased the endocytotic uptake of recombinant AGA in AGU patient cells. This effect was observed for the tagged (Strep‐tag, HQ‐tag) and the untagged AGA enzymes (Figure 7a). Competition experiments with 5 mM M6P led to a very profound, significant reduction of the endocytic uptake of AGA, and the S1S3 enhancing effect was completely lost (Figure 7b). Although S1S3 phosphotransferase is able to increase the M6P content of AGA, its co‐expression had no significant influence on the AGA enzyme processing (Figure 7c) or activity (Figure 7d) of the tagged AGA proteins.

### 3.6 N‐Glycosylation of Both AGA Subunits Is Required for Optimal M6‐Phosphorylation and Endocytic Uptake

**FIGURE 8:**
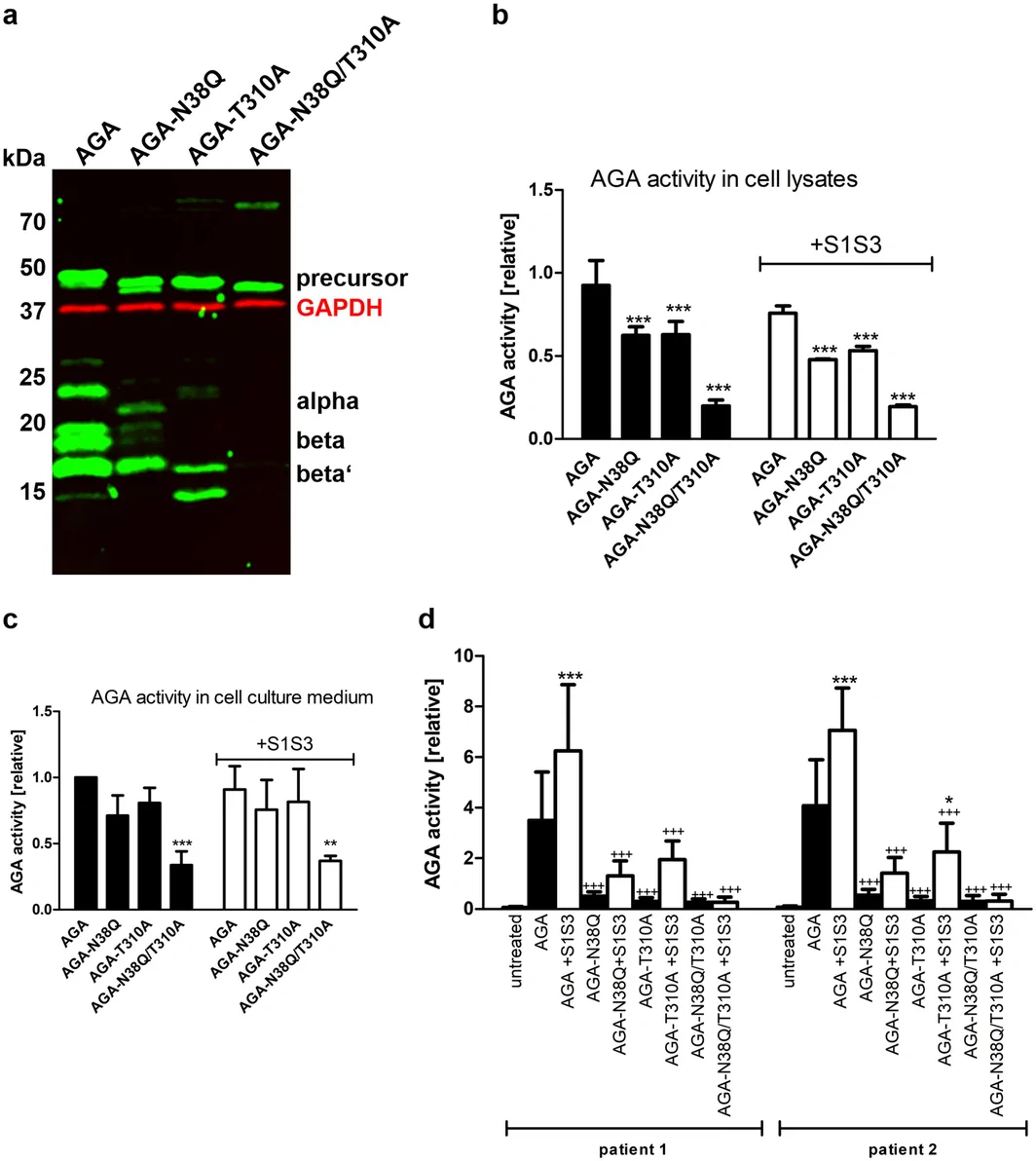
Influence of N‐glycosylation on recombinant AGA uptake. (a) AGA expression and processing of the N‐glycosylation mutants was analyzed by Western blotting. Size differences are due to the missing glycosylation of the AGA alpha subunit, in position Asn38 (N38Q), or the beta subunit in Asn308 (mutated as Thr310Ala = T310A). (b, c) Relative AGA activity in the cell lysates (b) and medium (c) of cells overexpressing wildtype AGA or the N‐glycosylation mutants with and without S1S3 coexpression was measured. (d) Wildtype AGA or AGA variants with mutated N‐glycosylation sites of the alpha subunit (N38Q), the beta‐subunit (T310A), or both subunits (N38Q + T310A) were overexpressed in the presence or absence of S1S3. The medium of the overexpressing cells was collected, and equal amounts of AGA activity were used for the treatment of AGU patient fibroblasts. Relative AGA activity in patient cell lysates was measured after 24 h of treatment. *n* = 3; two‐way ANOVA vs. wildtype AGA (symbol^+++^) or vs. respective AGA without S1S3 co‐expression (symbol ***). *p < 0.05, **p < 0.01, ***p < 0.001 for panels b–d, ^+++^ *p* < 0.001 for panel d.

AGA has two N‐glycosylation sites, one in each subunit [21]. To investigate whether the phosphorylation of both N‐glycans is required for the uptake and activity of AGA, the glycosylation sites of the alpha and beta subunits were mutated individually or in combination. The N‐glycosylation of the alpha subunit was prevented by the substitution Asn38Gln, whereas in the beta subunit, Thr310Ala substitution was produced, as Asn308Gln exchange results in an inactive and unstable AGA protein [21]. The lack of N‐glycans was visible as altered size patterns of the AGA subunits in Western blot (Figure 8a). The wildtype AGA showed a signal for the precursor (42 kDa), the alpha subunit at 24 kDa, and several signals between 15 and 20 kDa for the beta/beta’ subunit. The Asn38Gln variant exhibited a reduced size of the alpha subunit, whereas in the Thr310Ala variant, the beta subunit was smaller than in the wildtype AGA, due to the lack of N‐glycans. In addition, both variants showed a reduced size of the AGA precursor. If both N‐glycosylation sites were mutated simultaneously, AGA processing into subunits was severely reduced, and the Asn38Gln/Thr310Ala AGA precursor exhibited a lower molecular weight, as compared to the wildtype and the single glycosylation mutants (Figure 8a).

The reduced processing of the Asn38Gln/Thr310Ala double mutant was also evident in AGA enzyme activity measurements, for which the glycosylation site mutants Asn38Gln, Thr310Ala and Asn38Gln/Thr310Ala were overexpressed in HEK293T cells, together with S1S3 phosphotransferase or empty vector (Figure 8b,c). While the Asn38Gln and Thr310Ala single mutants exhibited about 75% AGA activity in cell lysates, the AGA activity of the double mutant was reduced to approximately 25% (Figure 8b). AGA with impaired N‐glycosylation of either subunit also seems to be secreted to a higher degree, instead of reaching lysosomes, as evidenced by comparison of AGA activity in cell lysates and medium (Figure 8b,c). Intracellularly, significantly less activity of the Asn38Gln‐ or Thr310Ala‐mutated AGA was present, as compared to the wildtype AGA, but this difference to the wildtype was non‐significant in the case of the culture medium (Figure 8b,c).

To study the endocytic uptake of the AGA variants, AGU patient fibroblasts were treated with conditioned medium containing the glycosylation deficient variants (Figure 8d). Patient fibroblasts were treated with the same amount of absolute AGA activity, determined by measuring the AGA activity in the conditioned medium before applying it to the cells. The endocytic uptake of the non‐mutated, wildtype AGA was significantly increased by co‐expression of S1S3, and this increase was also observed after mutation of either one of the two N‐glycosylation sites (Figure 8d). However, the baseline endocytic AGA uptake was much lower for the glycosylation site mutants than for the wildtype AGA enzyme. Importantly, if the N‐glycans on both the alpha and the beta subunits are missing, AGA can only poorly enter the cell, and S1S3 co‐expression does not result in an enhanced uptake (Figure 8d). This indicates that for the optimal endocytic uptake of AGA, both subunits should be glycosylated and modified with M6P, but AGA can also enter the cell with only one subunit carrying an M6‐phosphorylated N‐glycan, albeit with much lower efficiency.

## 4 Discussion

We have here shown that active, recombinant human AGA can efficiently be produced in eukaryotic cells and purified from the cell culture media, facilitating the development of ERT for AGU. So far, a high‐level production of AGA has not been feasible, as the inefficient processing/activation of the recombinant AGA was a major obstacle for producing amounts required for human ERT. Furthermore, purification of an untagged AGA is a complicated multi‐step process that requires the use of numerous chromatography methods [43]. In our study, we have been able to overcome these problems by constructing an affinity tagged AGA that is still efficiently processed into its active form and secreted into the cell culture medium, facilitating its purification in large amounts using simple one‐step affinity chromatography. After removal of the tag, the recombinant AGA protein is virtually identical to the endogenous one. Hence, the basic requirement for the development of an ERT for AGU using recombinant AGA is fulfilled.

AGA can be tagged at the C‐terminus without loss of activity, processing and uptake in patient cells. We used the Twin‐Streptag for the purification on a laboratory scale, and a complete removal of the tag is possible by TEV protease, after introducing the respective cleavage site N‐terminally to the Twin‐Streptag. However, entry into lysosomes and the C‐terminal processing of the beta subunit into the beta’ form would result in loss of the tag and in an inefficient purification. As an alternative to the C‐terminal tagging, an affinity tag can also be inserted directly after the AGA signal peptide, and the tag is then located at the beginning of the alpha subunit of AGA. Here, we have used a 12 amino acid long HQ‐tag, so that the tagged fusion proteins can be purified using metal affinity chromatography [44], and the tags can be subsequently removed [45]. For this, dipeptidyl aminopeptidase (DAPase) can be used, as it removes dipeptides from the amino terminus of a polypeptide until it encounters a dipeptide that it cannot remove, a so‐called stop point. Since AGA contains an intrinsic DAPase stop point, the complete HQ‐tag can be proteolytically removed without inserting any additional cleavage sites, resulting in an AGA enzyme that is identical to the natural one.

As soon as the lysosomal enzymes reach lysosomes, the M6P residues are removed by the lysosomal acid phosphatases Acp2 and Acp5, and in CHO cells, in which both Acp2 and Acp5 were knocked out, significantly higher levels of M6P proteins were found [46]. However, since we prefer the purification of overexpressed AGA from the cell culture medium, and the secreted AGA would not come in contact with the acid phosphatases, the avoidance of such phosphatases is not necessary. Therefore, purification of AGA from the cell culture medium should be preferred over purification from the lysates.

We here used HEK293T cells for AGA production, due to their simple cultivation, and were already able to achieve a high recombinant AGA protein expression. Due to the high degree of overexpression, most of the AGA is secreted and found in the cell culture medium. HDAC inhibitors, such as valproic acid, increased the expression and the amount of active recombinant AGA in the medium. The observed HDAC effects are not specific for AGA, but have been shown for example, for monoclonal antibody production in HEK293E and CHO‐DG44 cells [47, 48]. Valproic acid could potentially also be used for the production of further recombinant proteins for ERT. However, high concentrations of valproate resulted in an increased cell death. Therefore, the optimal concentration of valproate that allows the maximal expression with tolerable loss of cells should be determined.

It is plausible that the AGA protein yield could be further increased and the purification from the medium facilitated by using cell lines that grow under serum‐free conditions in suspension, such as HEK293‐F, or such that have been optimized for protein production on an industrial scale, such as Chinese hamster ovary cells (CHO). These cells are currently used for the production of a majority of therapeutic proteins, most of which are monoclonal antibodies (for review see [49]). However, due to such high rates of overexpression, it is likely that the overexpressed lysosomal proteins are sub‐optimally labeled with M6P. As insufficient M6P labeling appears to be a common problem for lysosomal ERT products, different strategies have been developed to increase the M6P content of therapeutic proteins (reviewed in [50]). For example, Alglucosidase alfa, an early ERT product for the treatment of Pompe disease, exhibited only low M6P levels, but the problem was overcome by glycoengineering of the optimized product, avalglucosidase alfa [33]. In addition to a direct labeling of the lysosomal proteins themselves, M6P labeling of liposomes or nanoparticles that contain the lysosomal protein is also conceivable. Such approaches are discussed for the targeted application of drugs in MPR300‐overexpressing tumors [51] and for the lysosomal degradation of extracellular or membrane proteins by lysosome‐targeting chimeras (LYTACs) that contain an antibody for the binding to the protein of interest, and an M6P‐modified part for binding to MPR300 [52].

The functionality of the S1S3 phosphotransferase has been successfully proven in a preclinical study for a hexosaminidase product (i.e., HexM) that could be used for the treatment of GM2 gangliosidosis [53], and for alpha‐N‐acetylglucosaminidase for Sanfilippo syndrome type B [54]. In a direct comparison of avalglucosidase alfa, which contains chemically coupled M6P glycans, with uncoupled alglucosidase alfa or alpha‐glucosidase expressed in the presence of S1S3, the S1S3 modified product was significantly better than alglucosidase alfa and avalglucosidase alfa in reducing the accumulated glycogen in a Pompe mouse model [55]. Furthermore, as S1S3 is less than half the size of the natural GlcNAc‐1‐phosphotransferase, it would be possible to use it for an AAV‐mediated gene therapy for patients suffering from mucolipidosis MLII/III, as has been shown in an animal model of MLII [56].

Inspired by the findings showing the benefits of increased M6‐phosphorylation, we generated a codon‐optimized version of S1S3, co‐expressed it with AGA, and were able to confirm the positive effect of S1S3 on M6‐phosphorylation and uptake of AGA. Our findings suggest that the lysosomal transport of AGA is largely dependent on M6P, since competition by M6P or mutation of both N‐glycosylation sites almost completely prevented the endocytic uptake of recombinant AGA. Although an alternative biosynthetic transport route for AGA from the ER to the lysosome was discussed in earlier studies [21], it does not appear to be of importance for the endocytic uptake of recombinant AGA into patient fibroblasts.

Due to the long half‐life and stability of AGA, the recombinant protein is well suitable for ERT. AGA is readily taken up by an M6P‐dependent route, and increasing the M6P content of AGA by coexpression of S1S3 increased its cellular uptake. The CI‐MPR is mainly responsible for the endocytosis of lysosomal enzymes, and it has two binding sites for M6P. Hence, bis‐phosphorylated N‐glycans have a much higher binding affinity than mono‐phosphorylated ones [57]. Thus, for ERT in AGU patients, the M6P content of the recombinant AGA should be optimized, and the application routes should be carefully considered, as it will be necessary to reach the brain for maximum benefit for the patients. Even though some steps in the production process of recombinant AGA still certainly require further optimization, and the recombinant protein needs to be studied in an animal model, our study is a major step toward an ERT for human AGU patients. Thus, after almost 60 years from the first description of AGU, we are one step closer to the development of a causal therapy for AGU.

## Author Contributions

Study design and supervision: Antje Banning and Ritva Tikkanen. Experimentation: Antje Banning, Adla Murad, and Lidia Reznikova. Visualization: Antje Banning and Ritva Tikkanen. Manuscript draft: Antje Banning and Ritva Tikkanen Manuscript editing: Antje Banning and Ritva Tikkanen.

## Funding

The authors have nothing to report.

## Ethics Statement

This study was approved by the Ethical Board of the Medical Faculty of the University of Giessen (Number 144/21). The parents of the AGU patient gave their written informed consent for the studies concerning the dermal fibroblasts.

## Conflicts of Interest

The authors declare no conflicts of interest.

## Data Availability

The data that support the findings of this study are available from the corresponding author upon reasonable request.

## References

[1] M. Arvio and I. Mononen , “Aspartylglycosaminuria: A Review,” Orphanet Journal of Rare Diseases 11 (2016): 162, 10.1186/s13023-016-0544-6. 27906067 PMC5134220

[2] K. Goodspeed , C. Feng , M. Laine , and T. C. Lund , “Aspartylglucosaminuria: Clinical Presentation and Potential Therapies,” Journal of Child Neurology 36 (2021): 403–414, 10.1177/0883073820980904. 33439067

[3] J. Saarela , M. Laine , C. Oinonen , et al., “Molecular Pathogenesis of a Disease: Structural Consequences of Aspartylglucosaminuria Mutations,” Human Molecular Genetics 10 (2001): 983–995. 11309371 10.1093/hmg/10.9.983

[4] K. J. Fisher and N. N. Aronson , “Characterization of the Mutation Responsible for Aspartylglucosaminuria in Three Finnish Patients. Amino Acid Substitution Cys163‐Ser Abolishes the Activity of Lysosomal Glycosylasparaginase and Its Conversion Into Subunits,” Journal of Biological Chemistry 266 (1991): 12105–12113. 1904874

[5] E. Ikonen , M. Baumann , K. Gron , et al., “Aspartylglucosaminuria: cDNA Encoding Human Aspartylglucosaminidase and the Missense Mutation Causing the Disease,” EMBO Journal 10 (1991): 51–58. 1703489 10.1002/j.1460-2075.1991.tb07920.x PMC452610

[6] I. Mononen , N. Heisterkamp , V. Kaartinen , et al., “Aspartylglycosaminuria in the Finnish Population: Identification of Two Point Mutations in the Heavy Chain of Glycoasparaginase,” Proceedings of the National Academy of Sciences of the United States of America 88 (1991): 2941–2945, 10.1073/pnas.88.7.2941. 2011603 PMC51356

[7] E. Ikonen , N. Enomaa , I. Ulmanen , and L. Peltonen , “In Vitro Mutagenesis Helps to Unravel the Biological Consequences of Aspartylglucosaminuria Mutation,” Genomics 11 (1991): 206–211. 1765378 10.1016/0888-7543(91)90120-4

[8] R. J. Pollitt , F. A. Jenner , and H. Merskey , “Aspartylglycosaminuria. An Inborn Error of Metabolism Associated With Mental Defect,” Lancet 2 (1968): 253–255. 4173687 10.1016/s0140-6736(68)92355-6

[9] M. Arvio , O. Sauna‐Aho , and M. Peippo , “Bone Marrow Transplantation for Aspartylglucosaminuria: Follow‐Up Study of Transplanted and Non‐Transplanted Patients,” Journal of Pediatrics 138 (2001): 288–290, 10.1067/mpd.2001.110119. 11174635

[10] T. Autti , J. Rapola , P. Santavuori , et al., “Bone Marrow Transplantation in Aspartylglucosaminuria: Histopathological and MRI Study,” Neuropediatrics 30 (1999): 283–288, 10.1055/s-2007-973506. 10706021

[11] G. Malm , J.‐E. Månsson , J. Winiarski , M. Mosskin , and O. Ringdén , “Five‐Year Follow‐Up of Two Siblings With Aspartylglucosaminuria Undergoing Allogeneic Stem‐Cell Transplantation From Unrelated Donors,” Transplantation 78 (2004): 415–419, 10.1097/00007890-200408150-00015. 15316370

[12] A. Selvanathan , J. Kinsella , F. Moore , et al., “Effectiveness of Early Hematopoietic Stem Cell Transplantation in Preventing Neurocognitive Decline in Aspartylglucosaminuria: A Case Series,” JIMD Reports 61 (2021): 3–11, 10.1002/jmd2.12222. 34485011 PMC8411101

[13] X. Chen , S. Snanoudj‐Verber , L. Pollard , et al., “Pre‐Clinical Gene Therapy With AAV9/AGA in Aspartylglucosaminuria Mice Provides Evidence for Clinical Translation,” Molecular Therapy 29 (2021): 989–1000, 10.1016/j.ymthe.2020.11.012. 33186692 PMC7934581

[14] U. Dunder , V. Kaartinen , P. Valtonen , et al., “Enzyme Replacement Therapy in a Mouse Model of Aspartylglycosaminuria,” FASEB Journal 14 (2000): 361–367, 10.1096/fasebj.14.2.361. 10657992

[15] U. Dunder , P. Valtonen , E. Kelo , and I. Mononen , “Early Initiation of Enzyme Replacement Therapy Improves Metabolic Correction in the Brain Tissue of Aspartylglycosaminuria Mice,” Journal of Inherited Metabolic Disease 33 (2010): 611–617, 10.1007/s10545-010-9158-7. 20607610

[16] E. Kelo , U. Dunder , and I. Mononen , “Massive Accumulation of Man2GlcNAc2‐Asn in Nonneuronal Tissues of Glycosylasparaginase‐Deficient Mice and Its Removal by Enzyme Replacement Therapy,” Glycobiology 15 (2005): 79–85, 10.1093/glycob/cwh145. 15342551

[17] M. Peltola , A. Kyttala , O. Heinonen , et al., “Adenovirus‐Mediated Gene Transfer Results in Decreased Lysosomal Storage in Brain and Total Correction in Liver of Aspartylglucosaminuria (AGU) Mouse,” Gene Therapy 5 (1998): 1314–1321, 10.1038/sj.gt.3300740. 9930336

[18] S. Virta , J. Rapola , A. Jalanko , and M. Laine , “Use of Nonviral Promoters in Adenovirus‐Mediated Gene Therapy: Reduction of Lysosomal Storage in the Aspartylglucosaminuria Mouse,” Journal of Gene Medicine 8 (2006): 699–706, 10.1002/jgm.892. 16518877

[19] A. Banning , C. Gülec , J. Rouvinen , S. J. Gray , and R. Tikkanen , “Identification of Small Molecule Compounds for Pharmacological Chaperone Therapy of Aspartylglucosaminuria,” Scientific Reports 6 (2016): 37583, 10.1038/srep37583. 27876883 PMC5120323

[20] M. Laine , A. Banning , V. Sairanen , et al., “Pharmacological Chaperone Treatment With Cystadane for Aspartylglucosaminuria: An Open‐Label, Phase 1b/2, Clinical Trial,” Research Square (2026), 10.21203/rs.3.rs-8103849/v1.

[21] R. Tikkanen , N. Enomaa , A. Riikonen , E. Ikonen , and L. Peltonen , “Intracellular Sorting of Aspartylglucosaminidase: The Role of N‐Linked Oligosaccharides and Evidence of Man‐6‐P‐Independent Lysosomal Targeting,” DNA and Cell Biology 14 (1995): 305–312, 10.1089/dna.1995.14.305. 7710687

[22] R. Tikkanen , M. Peltola , C. Oinonen , J. Rouvinen , and L. Peltonen , “Several Cooperating Binding Sites Mediate the Interaction of a Lysosomal Enzyme With Phosphotransferase,” EMBO Journal 16 (1997): 6684–6693, 10.1093/emboj/16.22.6684. 9362483 PMC1170273

[23] T. Braulke , J. E. Carette , and W. Palm , “Lysosomal Enzyme Trafficking: From Molecular Mechanisms to Human Diseases,” Trends in Cell Biology 34 (2024): 198–210, 10.1016/j.tcb.2023.06.005. 37474375

[24] T. Braulke and J. S. Bonifacino , “Sorting of Lysosomal Proteins,” Biochimica et Biophysica Acta 1793 (2009): 605–614, 10.1016/j.bbamcr.2008.10.016. 19046998

[25] P. Ghosh , N. M. Dahms , and S. Kornfeld , “Mannose 6‐Phosphate Receptors: New Twists in the Tale,” Nature Reviews. Molecular Cell Biology 4 (2003): 202–212, 10.1038/nrm1050. 12612639

[26] R. J. Desnick and E. H. Schuchman , “Enzyme Replacement Therapy for Lysosomal Diseases: Lessons From 20 Years of Experience and Remaining Challenges,” Annual Review of Genomics and Human Genetics 13 (2012): 307–335, 10.1146/annurev-genom-090711-163739. 22970722

[27] S. Ellison , H. Parker , and B. Bigger , “Advances in Therapies for Neurological Lysosomal Storage Disorders,” Journal of Inherited Metabolic Disease 46 (2023): 874–905, 10.1002/jimd.12615. 37078180

[28] F. M. Platt , “Emptying the Stores: Lysosomal Diseases and Therapeutic Strategies,” Nature Reviews. Drug Discovery 17 (2018): 133–150, 10.1038/nrd.2017.214. 29147032

[29] A. Schulz , T. Ajayi , N. Specchio , et al., “Study of Intraventricular Cerliponase Alfa for CLN2 Disease,” New England Journal of Medicine 378 (2018): 1898–1907, 10.1056/NEJMoa1712649. 29688815

[30] M. Arends , M. Biegstraaten , C. Wanner , et al., “Agalsidase Alfa Versus Agalsidase Beta for the Treatment of Fabry Disease: An International Cohort Study,” Journal of Medical Genetics 55 (2018): 351–358, 10.1136/jmedgenet-2017-104863. 29437868 PMC5931248

[31] L. D. M. Pena , R. J. Barohn , B. J. Byrne , et al., “Safety, Tolerability, Pharmacokinetics, Pharmacodynamics, and Exploratory Efficacy of the Novel Enzyme Replacement Therapy Avalglucosidase Alfa (neoGAA) in Treatment‐Naïve and Alglucosidase Alfa‐Treated Patients With Late‐Onset Pompe Disease: A Phase 1, Open‐Label, Multicenter, Multinational, Ascending Dose Study,” Neuromuscular Disorders 29 (2019): 167–186, 10.1016/j.nmd.2018.12.004. 30770310

[32] I. Mononen , N. Heisterkamp , U. Dunder , et al., “Recombinant Glycosylasparaginase and In Vitro Correction of Aspartylglycosaminuria,” FASEB Journal 9 (1995): 428–433, 10.1096/fasebj.9.5.7896015. 7896015

[33] A. Anding , S. Kinton , K. Baranowski , et al., “Increasing Enzyme Mannose‐6‐Phosphate Levels but Not Miglustat Coadministration Enhances the Efficacy of Enzyme Replacement Therapy in Pompe Mice,” Journal of Pharmacology and Experimental Therapeutics 387 (2023): 188–203, 10.1124/jpet.123.001593. 37679046

[34] L. Liu , W.‐S. Lee , B. Doray , and S. Kornfeld , “Engineering of GlcNAc‐1‐Phosphotransferase for Production of Highly Phosphorylated Lysosomal Enzymes for Enzyme Replacement Therapy,” Molecular Therapy ‐ Methods & Clinical Development 5 (2017): 59–65, 10.1016/j.omtm.2017.03.006. 28480305 PMC5415318

[35] D.‐B. Oh , “Glyco‐Engineering Strategies for the Development of Therapeutic Enzymes With Improved Efficacy for the Treatment of Lysosomal Storage Diseases,” BMB Reports 48 (2015): 438–444, 10.5483/bmbrep.2015.48.8.101. 25999178 PMC4576951

[36] H. Li , B. Doray , B. C. Jennings , et al., “Structure of a Truncated Human GlcNAc‐1‐Phosphotransferase Variant Reveals the Basis for Its Hyperactivity,” Journal of Biological Chemistry 300 (2024): 107706, 10.1016/j.jbc.2024.107706. 39178950 PMC11418123

[37] M. Kapahnke , A. Banning , and R. Tikkanen , “Random Splicing of Several Exons Caused by a Single Base Change in the Target Exon of CRISPR/Cas9 Mediated Gene Knockout,” Cells 5 (2016): 45, 10.3390/cells5040045. 27983621 PMC5187529

[38] A. Banning , L. Hoeren , I. Atallah , et al., “Structural and Functional Characterization of N‐Glycanase‐1 Pathogenic Variants,” Cells 14 (2025), 10.3390/cells14131036. PMC12248763 40643555

[39] A. Banning , A. Zakrzewicz , X. Chen , S. J. Gray , and R. Tikkanen , “Knockout of the CMP‐Sialic Acid Transporter SLC35A1 in Human Cell Lines Increases Transduction Efficiency of Adeno‐Associated Virus 9: Implications for Gene Therapy Potency Assays,” Cells 10 (2021): 1259, 10.3390/cells10051259. 34069698 PMC8160606

[40] S. Mussche , B. Devreese , S. Nagabhushan Kalburgi , et al., “Restoration of Cytoskeleton Homeostasis After Gigaxonin Gene Transfer for Giant Axonal Neuropathy,” Human Gene Therapy 24 (2013): 209–219, 10.1089/hum.2012.107. 23316953

[41] A. Banning , J. F. König , S. J. Gray , and R. Tikkanen , “Functional Analysis of the Ser149/Thr149 Variants of Human Aspartylglucosaminidase and Optimization of the Coding Sequence for Protein Production,” International Journal of Molecular Sciences 18 (2017): 706, 10.3390/ijms18040706. 28346360 PMC5412292

[42] J. Schindelin , I. Arganda‐Carreras , E. Frise , et al., “Fiji: An Open‐Source Platform for Biological‐Image Analysis,” Nature Methods 9 (2012): 676–682, 10.1038/nmeth.2019. 22743772 PMC3855844

[43] R. Tikkanen , J. Rouvinen , A. Torronen , N. Kalkkinen , and L. Peltonen , “Large‐Scale Purification and Preliminary x‐Ray Diffraction Studies of Human Aspartylglucosaminidase,” Proteins 24 (1996): 253–258, 10.1002/(SICI)1097-0134(199602)24:2<253::AID-PROT12>3.0.CO;2-M. 8984501

[44] B. Godat , L. Engel , N. A. Betz , and T. M. Johnson , “Methods for the Purification of HQ‐Tagged Proteins,” Methods in Molecular Biology (Clifton, N.J.) 421 (2008): 151–168, 10.1007/978-1-59745-582-4_11. 18826054

[45] J. Arnau , C. Lauritzen , and J. Pedersen , “Cloning Strategy, Production and Purification of Proteins With Exopeptidase‐Cleavable His‐Tags,” Nature Protocols 1 (2006): 2326–2333, 10.1038/nprot.2006.388. 17406475

[46] T. Čaval , J. Zhu , W. Tian , et al., “Targeted Analysis of Lysosomal Directed Proteins and Their Sites of Mannose‐6‐Phosphate Modification,” Molecular & Cellular Proteomics: MCP 18 (2019): 16–27, 10.1074/mcp.RA118.000967. 30237200 PMC6317476

[47] G. Backliwal , M. Hildinger , I. Kuettel , F. Delegrange , D. L. Hacker , and F. M. Wurm , “Valproic Acid: A Viable Alternative to Sodium Butyrate for Enhancing Protein Expression in Mammalian Cell Cultures,” Biotechnology and Bioengineering 101 (2008): 182–189, 10.1002/bit.21882. 18454496

[48] S. Wulhfard , L. Baldi , D. L. Hacker , and F. Wurm , “Valproic Acid Enhances Recombinant mRNA and Protein Levels in Transiently Transfected Chinese Hamster Ovary Cells,” Journal of Biotechnology 148 (2010): 128–132, 10.1016/j.jbiotec.2010.05.003. 20510314

[49] M.‐E. Lalonde and Y. Durocher , “Therapeutic Glycoprotein Production in Mammalian Cells,” Journal of Biotechnology 251 (2017): 128–140, 10.1016/j.jbiotec.2017.04.028. 28465209

[50] J. Seo and D.‐B. Oh , “Mannose‐6‐Phosphate Glycan for Lysosomal Targeting: Various Applications From Enzyme Replacement Therapy to Lysosome‐Targeting Chimeras,” Animal Cells and Systems 26 (2022): 84–91, 10.1080/19768354.2022.2079719. 35784393 PMC9246025

[51] C. Minnelli , L. Cianfruglia , E. Laudadio , et al., “Selective Induction of Apoptosis in MCF7 Cancer‐Cell by Targeted Liposomes Functionalised With Mannose‐6‐Phosphate,” Journal of Drug Targeting 26 (2018): 242–251, 10.1080/1061186x.2017.1365873. 28795851

[52] S. M. Banik , K. Pedram , S. Wisnovsky , G. Ahn , N. M. Riley , and C. R. Bertozzi , “Lysosome‐Targeting Chimaeras for Degradation of Extracellular Proteins,” Nature 584 (2020): 291–297, 10.1038/s41586-020-2545-9. 32728216 PMC7727926

[53] G. Benzie , K. Bouma , T. Battellino , et al., “Increased Phosphorylation of HexM Improves Lysosomal Uptake and Potential for Managing GM2 Gangliosidoses,” BBA Advances 2 (2021): 100032, 10.1016/j.bbadva.2021.100032. 37082581 PMC10074939

[54] P. I. Dickson , S. Q. Le , A. Sorensen , et al., “Co‐Expression of S1S3 Phosphotransferase in Production Cell Line Improves Mannose 6‐Phosphorylation and Cellular Uptake of Alpha‐N‐Acetylglucosaminidase (Sanfilippo Syndrome Type B),” Molecular Genetics and Metabolism 135 (2022): S38, 10.1016/j.ymgme.2021.11.085.

[55] R. Gotschall , K. Gray , M. DiGruccio , et al., “M021: A Novel Drug Candidate for Pompe Disease,” Molecular Genetics and Metabolism 144 (2025): 108742, 10.1016/j.ymgme.2024.108742.

[56] P. I. Dickson , J. Srnak , S. Q. Le , et al., “Gene Therapy With AAV‐S1S3 Improves Disease in Mucolipidosis Type II Mice,” Molecular Genetics and Metabolism 141 (2024): 107805, 10.1016/j.ymgme.2023.107805.

[57] P. Y. Tong , W. Gregory , and S. Kornfeld , “Ligand Interactions of the Cation‐Independent Mannose 6‐Phosphate Receptor. The Stoichiometry of Mannose 6‐Phosphate Binding,” Journal of Biological Chemistry 264 (1989): 7962–7969. 2542254

